# Hyaluronan production increases the malignant properties of mesothelioma cells

**DOI:** 10.1054/bjoc.2001.1922

**Published:** 2001-08

**Authors:** Y Li, P Heldin

**Affiliations:** Department of Medical Biochemistry and Microbiology, Unit of Biochemistry, Uppsala University, Biomedical Center, Box 575S-751 23 Uppsala, Sweden

**Keywords:** malignant mesothelioma, hyaluronan synthase, hyaluronan receptor, tumour spreading

## Abstract

Malignant pleural mesotheliomas is in most cases associated with elevated amounts of hyaluronan. To investigate the importance of hyaluronan for the malignant properties of mesotheliomas, we have expressed murine hyaluronan synthase 2 (HAS2) in the non-hyaluronan producing mesothelioma cell line, Mero-25. We found that upon hyaluronan overproduction the mesothelioma cells changed their epitheloid character to a fibroblastic phenotype and were surrounded by pericellular matrices, the size of which correlated to the amount of synthesized hyaluronan. HAS2-transfected cells with the ability to synthesize about 520 ng hyaluronan/5 × 10^4^cells/24 h exhibited about a 2-fold increase in the expression of the cell surface hyaluronan receptor CD44 and their locomotion increased compared to that of mock-transfected Mero-25 cells. Furthermore, the malignant properties of mesothelioma cell clones as determined by the ability to grow in a soft agar assay correlated to their hyaluronan production. These results provide evidence for an important role of hyaluronan in the aggressive spread of mesotheliomas in adjacent non-cancerous stromal tissues. © 2001 Cancer Research Campaign http://www.bjcancer.com


*British Journal of Cancer* (2001) **85**(4), 600–607
© 2001 Cancer Research Campaign
doi: 10.1054/ bjoc.2001.1922, available online at http://www.idealibrary.com on IDEAL

http://www.bjcancer.com

### Hyaluronan production increases the malignant properties of mesothelioma cells

**Y Li[1] and P Heldin**

Department of Medical Biochemistry and Microbiology, Unit of Biochemistry, Uppsala University, Biomedical Center, Box 575, S-751 23 Uppsala, Sweden

**Summary** Malignant pleural mesotheliomas is in most cases associated with elevated amounts of hyaluronan. To investigate the importance of hyaluronan for the malignant properties of mesotheliomas, we have expressed murine hyaluronan synthase 2 (HAS2) in the non-hyaluronan producing mesothelioma cell line, Mero-25. We found that upon hyaluronan overproduction the mesothelioma cells changed their epitheloid character to a fibroblastic phenotype and were surrounded by pericellular matrices, the size of which correlated to the amount of synthesized hyaluronan. HAS2-transfected cells with the ability to synthesize about 520 ng hyaluronan/$5 \times 10^4$ cells/24 h exhibited about a 2-fold increase in the expression of the cell surface hyaluronan receptor CD44 and their locomotion increased compared to that of mock-transfected Mero-25 cells. Furthermore, the malignant properties of mesothelioma cell clones as determined by the ability to grow in a soft agar assay correlated to their hyaluronan production. These results provide evidence for an important role of hyaluronan in the aggressive spread of mesotheliomas in adjacent non-cancerous stromal tissues. © 2001 Cancer Research Campaign http://www.bjcancer.com

**Keywords**: malignant mesothelioma; hyaluronan synthase; hyaluronan receptor; tumour spreading

Hyaluronan is a linear glycosaminoglycan, which is ubiquitiously distributed in the extracellular matrix and regulates several normal physiological functions such as cell migration, growth, differentiation and cell adhesion (Laurent and Fraser, 1992; Laurent et al, 1995; Knudson, 1998). It is synthesized at the inner leaflet of the plasma membrane by hyaluronan synthases (HAS1, HAS2 and HAS3; Itano and Kimata, 1996; Shyjan et al, 1996; Spicer et al, 1996, 1997; Watanabe and Yamaguchi, 1996); each HAS isoform is capable of synthesizing hyaluronan, exhibits a cell specific expression pattern, and possesses distinct enzymic properties (Brinck and Heldin, 1999; Itano et al, 1999b; Jacobson et al, 2000). Among the three HAS isoforms tested it appears that mainly HAS2 expression is affected in response to external stimuli (Brick and Heldin, 1999; Li et al, 2000; Jacobson et al, 2000).

Several studies have shown that hyaluronan synthesis is often increased in certain human tumours and occur predominantly within the tumour-adjacent connective tissue (Bertrand et al, 1992; Auvinen et al, 1997; Ropponen et al, 1998). The hyaluronan in tumours is either produced by tumour cells themselves (Delpech et al, 1997), or by adjacent noncancerous stromal cells (Asplund et al, 1993). Hyaluronan overproduction leads to remodelling of the extracellular matrix surrounding tumour cells and correlates with their invasive and metastatic behaviour (Kimata et al, 1983; Zhang et al, 1995).

Mesothelioma is a malignant tumour of the pleura and peritoneum which is linked to asbestos exposure. However, other factors such as chemical carcinogens, chronic inflammation and viral SV 40 exposure may contribute to the development of this neoplasia (Bielefeldt-Ohmann et al, 1996). Mesotheliomas do not exhibit abnormalities in the common target genes *ras* and *p53*, but loss of *p16INK4a* expression is commonly observed in these tumours (Metcalf et al, 1992; Frizelle et al, 1998). Malignant mesothelioma is characterized by a highly invasive behaviour (Scarpa et al, 1999). A number of mesotheliomas produce hyaluronan (Klominek et al, 1989), whereas others do not produce hyaluronan but release growth factors which stimulate surrounding normal cells to synthesize hyaluronan (Asplund et al, 1993).

In this study, we investigated the relationship between cellular hyaluronan production and the malignant phenotype of mesothelioma cells. We transfected the non-hyaluronan producing mesothelioma cell line Mero-25 (Asplund *et al*, 1993) with cDNA for HAS2, and demonstrated that hyaluronan production by Mero-25 cells induced differentiation of the cells to a more fibroblastic phenotype. Furthermore, increased hyaluronan synthesis enhanced cell proliferation, anchorage-independent growth and cell migration.

### MATERIALS AND METHODS

### Materials and cell lines

Ham's F-10 medium and fetal calf serum (FCS) were purchased from Swedish Veterinary Institute (Uppsala, Sweden). Opti-MEM medium and LipofectAMINE were purchased from Gibco, UK. G418 sulfate (geneticin) was purchased from Calbiochem, CA. Vitrogen 100 (purified type I collagen) was purchased from Collagen Corporation (Palo Alto, CA). pCIneo plasmid containing open reading frame (ORF) for mouse HAS2 was a generous gift from Dr Andrew Spicer, California, USA. The human mesothelioma cell line, Mero-25, was kindly provided by Dr M Versnel (Rotterdam, Netherlands). *Streptomyces* hyaluronidase was purchased from Seikagaku Corporation, Japan. [$^3$H]glycosamine hydrochloride (18.5 Ci mmol$^{-1}$) was purchased from DuPont NEN

*Received 2 January 2001*
*Revised 24 April 2001*
*Accepted 26 April 2001*

*Correspondence to:* P Heldin

[1]Present address: Institute of Radiation Medicine, 27#, Tai Ping Road, Beijing, China.

(Boston, MA). [3]H-labelled hyaluronan with a specific activity of $42 \times 10^4$ dpm $\mu g^{-1}$, and a molecular weight of $3.85 \times 10^6$ was generously provided by Dr JRE Fraser (Melbourne, Australia). Hyaluronan of various molecular weights was a kind gift from Dr O Wik (Q-Med, Uppsala, Sweden). The human CD44 mAb against Hermes-3 (mouse IgG2a) was kindly provided by Dr S Jalkanen (Turku, Finland). Cyclin A, cyclin B, p21 and Cdc2 p34 monoclonal antibodies were purchased from Santa Cruz (UK). Secondary peroxidase-conjugated anti-mouse IgG, were purchased from Amersham Pharmacia Biotech (Sweden).

### Stable transfection of HAS2 cDNA into mesothelioma cells

The human mesothelioma cells, Mero-25, were grown in Ham's F-10 medium containing 15% FCS, 4 mM glutamine, 100 IU $ml^{-1}$ penicillin and 100 $\mu g$ $ml^{-1}$ streptomycin (complete medium). Subconfluent cultures of Mero-25 cells were transfected with 2 $\mu g$ $ml^{-1}$ of pCIneo vector containing the open reading frame for HAS2 (Spicer et al, 1996) using LipofectAMINE according to the manufacturer's instructions. Mock-transfected cells, i.e. cells transfected with the empty pCIneo vector (Promega), were used as a control. 3 days after transfection, single clones were isolated with a pipette and seeded on 24-well plates in complete medium supplemented with 800 $\mu g$ $ml^{-1}$ G418 for selection of stable transfected clones. G418-resistant colonies were examined for HAS2 expression by measuring the hyaluronan content in 24 h conditioned media by using a commercial kit (Pharmacia HA Test, Uppsala, Sweden). This kit is based on the specificity of a hyaluronan-binding protein (Tengblad, 1980).

### Determination of hyaluronan synthesized by mesothelioma transfectants

The hyaluronan-synthesizing capacity of HAS2-transfectants was determined by visualization of hyaluronan containing pericellular coats essentially as descibed previously (Clarris and Fraser, 1968; Heldin and Pertoft, 1993). Sparsely seeded mock-and HAS2-transfectant clones ($5 \times 10^4$ cells/35 mm dish) were incubated for 24 h in complete medium, and were then overlaid with $1 \times 10^7$ formalin-fixed erythrocytes. The erythrocytes were then allowed to settle for 15 min at room temperature and the cells were observed with an inverted microscope with a phase contrast at 600x magnification with a Nikon camera. The size of the pericellular coat was defined by the ratio between the area excluding erythrocytes and cell area and was calculated by using NIH image program. A ratio of the excluded area:cell area equal to 1.0 represents no detectable coats.

To determine the molecular mass of hyaluronan molecules synthesized de novo by mock-transfected cells or cells stably transfected with cDNA for HAS2, $1 \times 10^5$ cells $well^{-1}$ on a 6-well plate were grown in 1 ml complete medium supplemented with 5 $\mu Ci$ of [$^3$H]glucosamine, a precursor of both hyaluronan and sulfated glycosaminoglycans. After 24 h, conditioned media from 2 wells were harvested, combined and dialysed for 48 h against 0.1 M sodium acetate buffer, pH 5.5, containing 5 mM EDTA ($M_w$ cut-off 3500). The dialysate was then divided into 2 equal aliquots. One aliquot was treated overnight with 10 U $ml^{-1}$ *Streptomyces* hyaluronidase at 37˚C, whereas the other was treated in a similar way but in the absence of the hyaluronidase. Following enzyme inactivation by heating, the samples were subjected to gel chromatography on a Sephacryl-HR column (1 × 100 cm) composed of different layers of Sephacryl-HR with different porosities (Lebel et al, 1988). The column was equilibrated and eluted with 0.25 M NaCl containing 0.05% chlorbutanol. Fractions of 1 ml were collected and the radioactivity was measured in a Pharmacia LKB Wallac scintillation counter.

### Proliferation assay

Differencies in the proliferative capacity between HAS2- and mock-transfectants were examined by counting the cell number at 1, 3, 6 and 9 days after subculturing. The relative increase in the cell number was defined by the ratio between the amount of cells at different times after subculture and the cell number obtained at 1 day after subculture.

### Cell cycle analysis

HAS2- or mock-transfected mesothelioma cells ($1 \times 10^5$ cells/well in 6-well plates) were cultured in complete medium for 24 h. Then, the cells were synchronized by culturing in starvation medium for an additional 48 h. At this time, the starvation medium was aspirated and fresh medium containing 3% FCS was added. After 48 h of culturing, cells were washed in PBS, trypsinazed and pelleted by centrifugation. For flow cytometric analysis, the cell pellets ($1 \times 10^6$ cells) were resuspended in 450 $\mu l$ stock solution buffer (3.4 mM trisodium citrate, 0.1% Nonidet P 40, 1.5 mM spermine tetrahydrochloride and 0.5 mM Tris, pH 7.6) containing trypsin (15 $\mu g$ $ml^{-1}$). Cells where then subjected to DNA content analysis and cell cycle kinetics as described by Vindelöv (Vindelöv et al, 1983) using a Becton Dickinson FACStar plus flow cytometer.

### Soft agar growth assay

Mock- and HAS2-transfected mesothelioma cells ($1.5 \times 10^5$ cells) were separately suspended in 3 ml F-10 medium containing 15% FCS prewarmed to 37˚C. Each cell suspension was mixed with 300 $\mu l$ of a prewarmed (60 ˚C) 3% agarose/PBS solution and layered into 3 wells of 6-well plates (1 ml $well^{-1}$), that were previously coated with 1 ml of 0.6% agarose in F-10 medium. The agarose was allowed to solidify at room temperature for 20 min before 3 ml of complete medium was added to each well. At 4 weeks plating, 10 fields were selected randomly and colonies larger than 0.5 arbitrary units were counted by a Nikon microscope. The area of the colonies was measured by using a NIH image program.

### Western blotting

Cell lysates ($1 \times 10^6$ cells per 100-mm dish) were solubilized in ice-cold RIPA buffer (10 mM Tris-HCl, pH 7.4, 0.15 M NaCl, 1% Triton X-100, 0.1% SDS, 0.65 mM $MgSO_4$ 1 mM $CaCl_2$, 0.5% deoxycholate) containing protease inhibitors (10 turbidity units $ml^{-1}$ aprotinin, 1 $\mu g$ $ml^{-1}$ leupeptin, 0.1 mM pefabloc, 1 $\mu M$ pepstatin and 0.25 mg $ml^{-1}$ DNase I) for 2 h at 4˚C, using a rocking platform. Lysates were centrifuged at 13 000 rpm for 15 min at 4˚C and the protein content of the supernatants was determined using a protein assay kit (Bio-Rad Laboratories). Equal amounts of protein (30 $\mu g$) were subjected to 10% SDS-PAGE and the protein was then electrophoretically transferred to nitrocellu-

*© 2001 Cancer Research Campaign* 

lose membranes. Non-specific binding sites on membranes were blocked overnight at 4˚C with 5% defatted milk in Tris-buffered saline (TBS; 20 mM Tris/HCl, 137 mM NaCl, pH 7.6) supplemented with 0.1% Tween 20. The membranes were then probed either with a mAb against Hermes-3 (10 µg $ml^{-1}$), or with mAbs against p21, cyclin A, cyclin B and Cdc2 p34 (2 µg $ml^{-1}$) for 3 h at room temperature. After three washes with TBS-Tween, the membranes were incubated with horseradish peroxidase-conjugated anti-mouse IgG for 1 h, and immunocomplexes detected by enhanced chemiluminescence (Amersham, Corp) according to the manufacturer's instructions. The blots were quantified using a scanner and associated software (Molecular analyst, BioRad).

### Chemotaxis assays

The migration capacities of mock-and HAS2-transfected cells were assayed in a Boyden chamber. Subconfluent cultures were harvested, resuspended in PBS containing 0.25% BSA and the cell number was adjusted to $2 \times 10^5$ cells $ml^{-1}$. Then the wells in the upper compartment were loaded with 50 µl of the cell suspension; chemoattractant in the lower chamber, Ham's F-10 medium containing 1% FCS was added. In another experiment, cells were added to the wells in the upper chamber, and as chemoattractant, hyaluronan of a molecular weight of $3.9 \times 10^6$ was used at different concentrations. The 2 chambers were separated by a 8 µm thick polycarbonate filter with 8 µm pores (Corning Costar, Netherlands), precoated overnight with a mixture of 100 µg $ml^{-1}$ vitrogen and 2 µg $ml^{-1}$ fibronectin solution in PBS at 4˚C. The migration assays were run for 4.5 h at 37˚C in a humidified atmosphere of 5% $CO_2$. At the end of the assay, the filters were removed, fixed in ice-cold methanol, stained with Giemsa stain solution and the cells on the upper side of the filter removed by scraping with a cotton swab. The filters were then mounted between glass slide and coverslip with Mountex. The number of cells per well that had migrated through pores to the lower side of the filter were determined by counting the stained cells under a light microscope at a magnification of 480x. For each set of experiments, the migration capacity of mock-transfected cells toward 1% FCS or toward PBS containing 0.25% BSA was used as a control and was referred to as 100% migration. All experiments were performed in triplicates and repeated 3 times.

### Hyaluronan-binding assay

The binding assay using [$^3$H]hyaluronan was performed essentially as described by Asplund and Heldin (Asplund and Heldin, 1994). Briefly, subconfluent cultures of mock-and HAS2-transfected cells were cultured overnight in F-10 medium containing 0.1% FCS (starvation medium) to remove traces of serum-derived hyaluronan. However, in order to ensure that the hyaluronan-binding sites were not occupied by endogenously synthesized hyaluronan, the cell cultures were also treated with testicular hyaluronidase. Following harvesting, the cells were aliquoted ($5 \times 10^5$ cells tube) in duplicate into BSA-precoated Eppendorf tubes and various concentrations of [$^3$H]hyaluronan, in the absence or presence of nonlabelled hyaluronan (100 µg $ml^{-1}$; $M_r$ $1.2 \times 10^6$), were added and the cells were incubated for 60 min at room temperature with gentle shaking. Following washing with PBS-D the cultures were solubilized in 0.3 M NaOH-1% SDS (w/v) for 30 min at room temperature. After neutralization with 2 M HCl, the suspension was mixed with scintillation cocktail (Ready Safe; Beckman) and subjected to scintillation counting. Specific binding was determined by subtraction of nonspecific binding (label retained after the addition of nonlabelled hyaluronan).

### Statistics

All statistical comparisons were made using paired Student's *t*-test. All statistic calculations were performed with Stat View 4.01.

### RESULTS

### Synthesis of hyaluronan by HAS2-transfected Mero-25 cells

In order to investigate whether hyaluronan production affects the malignant phenotype of mesotheliomas, Mero-25 cells were stably transfected with cDNA for HAS2. After selection with G418, 15 of the drug-resistant clones were isolated and established as stable cell lines. The expression of HAS2 in transfected cell clones was examined indirectly by quantification of hyaluronan content in conditioned media. Among the G418 resistant clones, 3 clones exhibiting hyaluronan synthesizing capacity were choosen for further experiments. The 3 clones, designated M25-13, M25-15 and M25-03, synthesized about 300 ng, 520 ng and 840 ng hyaluronan per $5 \times 10^4$ cells and 24 h, respectively. A mock-transfected Mero-25 cell clone which produced only minute amounts of hyaluronan, was designated MV-01 and used as a control (Table 1).

The size of hyaluronan chains synthesized de novo by M25-03, the transfectants producing the highest amount of hyaluronan, was determined by gel chromatography (Figure 1). The labelled hyaluronan synthesized by M25-03 cells eluted in the void volume of a Sephacryl column corresponding to a size larger than $3.9 \times 10^6$. A second peak of an average molecular weight of $4 \times 10^4$ was insensitive to *Streptomyces* hyaluronidase treatment and was therefore considered to represent other glycosaminoglycans. Both mock- and HAS2-transfectants synthesized similar amounts of non-hyaluronan glycosaminoglycans (Figure 1). The other 2 transfectants, M25-13 and M25-15, also synthesized large hyaluronan chains (data not shown). The size of hyaluronan synthesized by normal mesothelial cells was also studied and found to be of high molecular mass (data not shown).

### Overexpression of HAS2 gene affects cell morphology and size of pericellular coats

Untransfected or mock-transfected Mero-25 mesothelioma cells exhibit an epithelial-like phenotype. After transfection with cDNA for HAS2, a morphological conversion was observed; HAS2-transfectants exhibited a more fibroblastic morphology than mock-transfected cells which retained their epithelial character (Figure 2). This epitheloid morphology of mock-transfectants was not altered even if the cells were cocultured with exogenous hyaluronan (4 µg $ml^{-1}$) in a mechanically induced wounding experiment in vitro (Brinck and Heldin, 1997; data not shown). The fibroblastic morphology of HAS2-transfected cells was more apparent at subconfluence than at confluence (data not shown).

Our previous studies revealed that mesothelial cells produce large amounts of hyaluronan and are surrounded by hyaluronan containing coats. In contrast, mesothelioma cells only produce minute amounts of hyaluronan and do not form coats (Heldin et al, 1995). We therefore investigated whether HAS2-transfectants could

*© 2001 Cancer Research Campaign*

**Table 1** Hyaluronan production and pericellular coat forming capacity of mock- and Has2-transfectants

| Transfectants | Hyaluronan production (ng/5 × $10^4$ cells/24 h) | Area excluding erythrocytes / Cell area |
|---|---|---|
| MV-01 | 28 ± 2 | 1.00 ± 0.0 |
| M25-13 | 298 ± 12 | 2.10 ± 0.2[a] |
| M25-15 | 519 ± 59 | 2.62 ± 0.5[a] |
| M25-03 | 836 ± 9 | 2.97 ± 0.5[a] |

Hyaluronan accumulation in 24 h conditioned media was estimated by using a commercial kit; each value represents the average of duplicates ± range. Cells (5 × $10^4$ per 35 mm culture dish) were subjected to morphometric analysis after 24 h of culture in complete medium as described in Materials and Methods. The ratio between the area excluding erythrocytes and cell area are the mean values of 20 randomly choosen cells ± SEM.
[a]Compared to MV-01, $P < 0.05$.

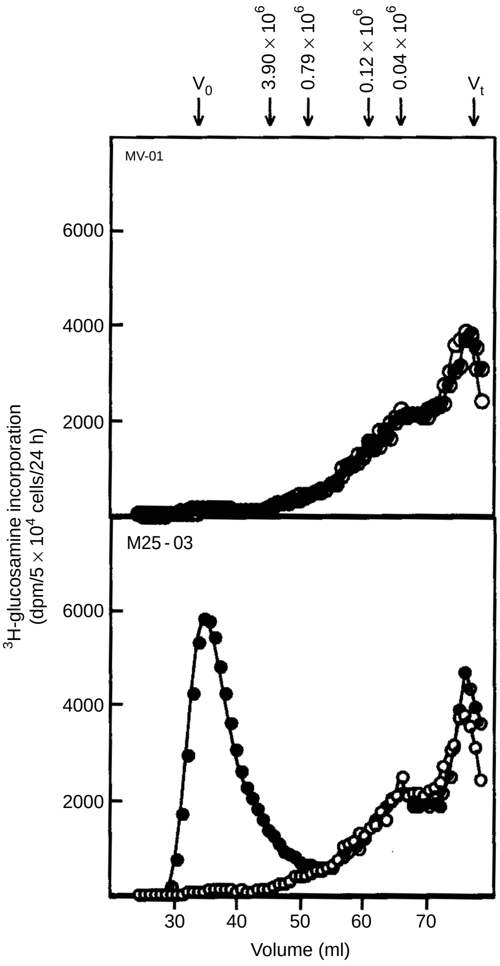

**Figure 1** Gel chromatography on a Sephacryl-HR column of glycosaminoglycans synthesized by HAS2 transfected mesothelioma cells. Mock (MV-01)- and HAS2 (M25-03)-transfected mesothelioma cells were labelled with [$^3$H]glucosamine for 24 h, and synthesized hyaluronan and other glycosaminoglycans were analysed on a Sephacryl-HR column (100 × 1 cm) as described in Materials and Methods. Closed and open circles represent untreated and *Streptomyces* hyaluronidase treated material, respectively. The peaks sensitive to hyaluronidase degradation represent newly synthesized hyaluronan. The elution positions of hyaluronan molecular weight markers are indicated by arrows form pericellular matrices. Mock-transfected cells did not form visible coats, whereas 3 clones with different hyaluronan synthesizing capacity were able to form coats. The size of the coats around HAS2-transfectants correlated well to the amounts of hyaluronan released into the medium from the individual transfectants (Table 1, Figure 2). These results indicate that the expression of HAS2 gene and subsequent synthesis of hyaluronan by mesotheliomas affect the cell shape and the organization of pericellular matrix, and thus the cellular functions, most likely through other mechanisms than the hyaluronan produced by neighbouring fibroblasts or mesothelial cells.

### Overproduction of hyaluronan makes cells more anchorage independent

We investigated the ability of mock- and HAS2-transfectants to proliferate in the absence of attachment to a solid substrate by using the soft agar assay; such anchorage-independent growth correlates well with the transformed phenotype in vivo (Freedman and Shin, 1974). The hyaluronan producing clones formed larger colonies in soft agar than mock-transfected cells (Figure 3). The area of colonies formed by the low (M25-13) and medium (M25-15) hyaluronan producing clones were about 2-fold higher than the mock-transfected cells (Figure 3B); an about 3-fold increase was detected in the high hyaluronan producing clone, M25-03. Furthermore, the number of the colonies was well correlated to the amounts of hyaluronan synthesized by HAS2-transfectants (data not shown). Moreover, the shape of the colonies formed by HAS2-transfected cells was more irregular than the untransfected cells (Figure 3A). These results suggest that increased hyaluronan production may enhance mesothelioma cell tumorigenicity.

Differencies in the proliferative capacity between mock- and HAS2-transfectants were investigated by calculating the cell number at different times after subculture. HAS2-transfected mesothelioma cells exhibited about a 2-fold higher proliferative capacity than mock-transfected cells (data not shown). The overexpression-induced enhancement of cell proliferation prompted us to investigate the expression levels of proteins involved in regulation of the cell cycle. We analysed cell cycle regulatory proteins by Western blot of asynchronously growing cells (Figure 4). The analysis revealed no significant changes in p21 and Cdc2 p34 expression but an up-regulation of the expression of cyclins A and B by the HAS2-transfectants. The increases of cyclins A and B may be related to the higher proliferative rate of hyaluronan-producing mesothelioma cells.

To assess further that HAS2-transfected mesothelioma cells proliferate more rapidly than mock-transfected cells, we analysed the cell cycle profiles of the hyaluronan producing clone M25-15 and mock-transfected clone MV-01 synchronized by serum-starvation using flow cytometry. 24 hours after release from quiencence by serum-stimulation, both the nonhyaluronan-producing and hyaluronan-producing clones began to move from the $G_1$ phase to S and $G_2$/M phase in a similar manner (data not shown). However, as shown in Figure 5, by 48 h after serum stimulation, HAS2-transfected cells had a higher proportion of cells in S phase (26.6%) compared to mock-transfected cells (18.9%) consistent with their faster proliferation.

### Overexpression of HAS2 induce cell motility and CD44 expression

We also examined if hyaluronan overproduction affected the migration of mesothelioma cells. The HAS2-transfected cells placed in the upper compartment of a Boyden chamber and exposed

© 2001 Cancer Research Campaign

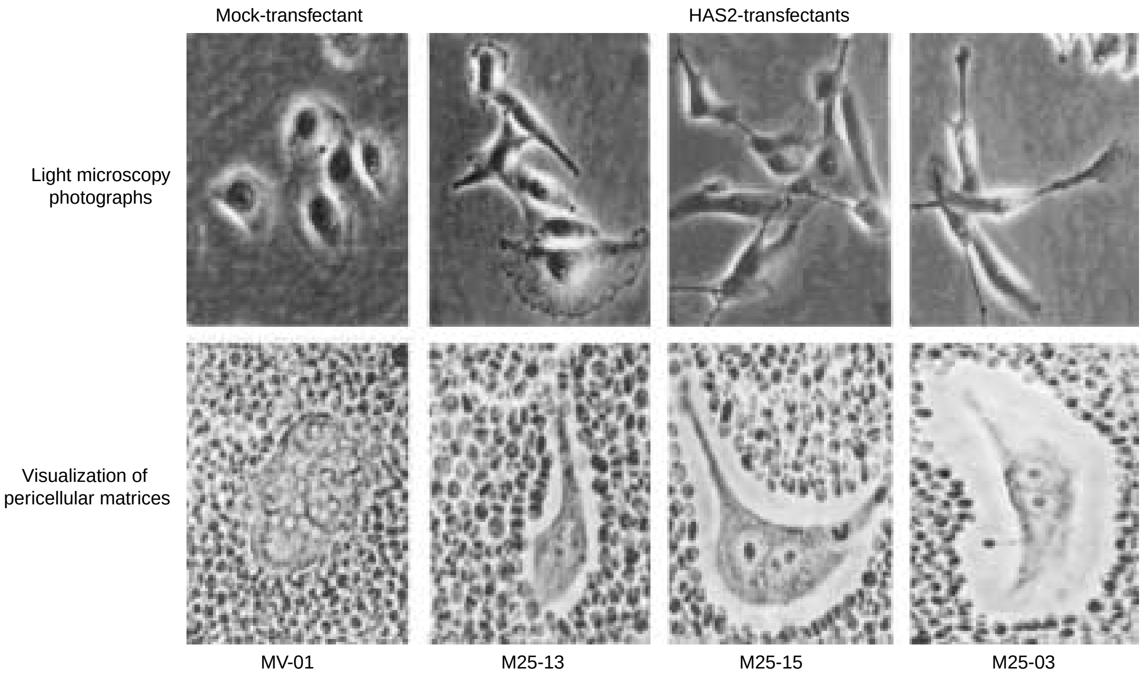

**Figure 2** HAS2 transfection induces morphological changes of mesothelioma cells and leads to formation of pericellular coats. Phase-contrast photographs of mock-transfected mesothelioma cells (MV-01) and HAS2-transfected mesothelioma clones producing different amounts of hyaluronan (M25-13, M25-15, M25-03), demonstrate that the HAS2-transfected cells exhibit a fibroblastic phenotype. In contrast, mock-transfected cells show an epithelial-like phenotype. Cells ($2 \times 10^4$ cells/35 mm dish) were cultured overnight in complete medium, then formalin fixed erythrocytes were added and the pericellular matrices were visualized, as described in Materials and Methods. Phase-contrast photographs of the clones demonstrate the effects of overproduction of hyaluronan on coat formation. A magnification of 600x is shown

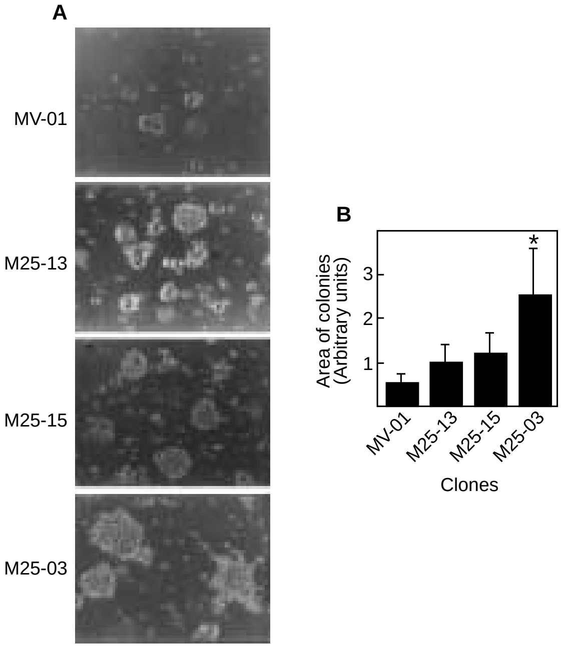

**Figure 3** Hyaluronan overproduction promotes soft agar colony formation. Phase contrast photographs (**A**) and area of colonies (**B**) of mock- and HAS2-transfected cells grown in soft agar, as described in Materials and Methods, are shown. The area of colonies larger than 0.5 arbitary units was calculated in 10 randomly selected fields/well using NIH image program; bars, SEM. The data shown are representative of 3 separate experiments

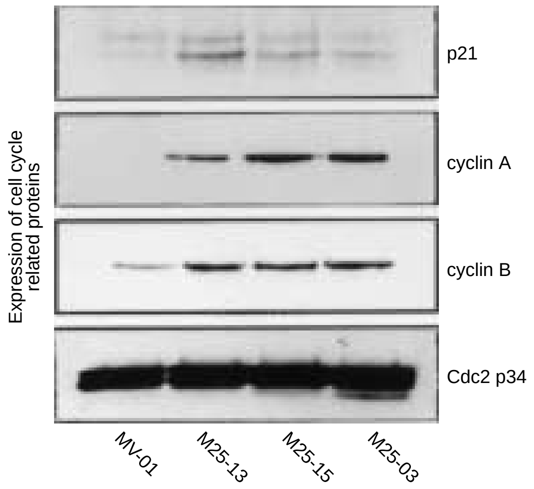

**Figure 4** Expression of p21, cyclin A, cyclin B and Cdc2 p34 in mock- and HAS2-transfected mesothelioma cells. Cell lysates (30 μg) from non-synchronized mock (MV-01)- and HAS2-transfectants (M25-13, M25-15, M25-03) were subjected to 10% SDS-PAGE, blotted onto nitrocellulose membrane, and probed with 2 μg $ml^{-1}$ of monoclonal antibodies against p21, cyclin A, cyclin B and Cdc2 p34. The data shown are representative of 3 different experiments to 1% FCS exhibited higher migratory rates compared to that of mock-transfected cells (Figure 6A). The M25-15 and M25-03 cells which synthesize about 520 ng and 840 ng hyaluronan per $5 \times 10^4$ cells and day respectively, migrated through the filter about 2-fold faster than the control MV-01 cells (Figure 6A).

Since HAS2-transfected mesothelioma cells produced hyaluronan of high molecular mass (more than $3.9 \times 10^6$ dalton), we also investigated if hyaluronan of a molecular weight of $3.9 \times 10^6$ affected the migration ability of mock-transfected mesothelioma

 *© 2001 Cancer Research Campaign*

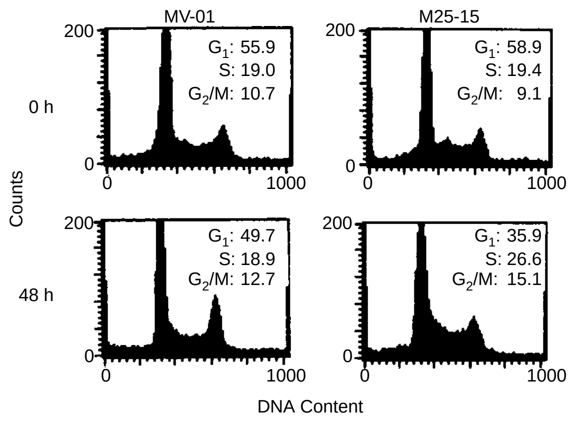

**Figure 5** Effects of overproduction of hyaluronan on the distribution of cells in different stages of the cell cycle. Synchronized mock (MV-01) and HAS2-transfected (M25-15) clones were analysed for their cell cycle profiles, as described in Materials and Methods. At 48 h after serum release, a higher portion of M25-15 cells were in S phase compared with MV-01. The data shown is representative of 3 separate experiments cells. As shown in Figure 6B, hyaluronan used as attractant in the lower compartment of the chamber stimulated in a dose-dependent manner the motility of MV-01 cells; a 2-fold increase in the migration was observed already at 10μg $ml^{-1}$ hyaluronan. The motile response did not increase considerably at higher hyaluronan concentrations.

CD44 is a ubiquitous multifunctional cell surface molecule which is the principle receptor for hyaluronan and is implicated in cell–cell and cell–extracellular matrix interactions, cell migration and lymph node homing (Naot et al, 1997; Knudson, 1998). Therefore, we investigated CD44 expression and hyaluronan binding capacity. As shown in Figure 7A, hyaluronan producing clones exhibited about a 2-fold higher expression of CD44 molecules compared to that of MV-01 control cells. Only the CD44 variant with a molecular mass of about 90 kDa was expressed. Furthermore, we investigated whether this induction of CD44 molecules was associated with a comparable increase in specific hyaluronan binding sites on the cell surface of M25-15 clone (Figure 7B). The M25-15 cells exhibited about a 2-fold more specific binding sites for [$^3$H]hyaluronan than the MV-01 cells. Interestingly, the degree of the increased CD44 expression and hyaluronan-binding capacity correlated well with the production of hyaluronan by the cell clones tested.

### DISCUSSION

Malignant mesothelioma is histologically classified into 3 main variants: epitheloid, fibrous and biphasic. Clinically, the epitheloid histotype is the least aggressive, followed by biphasic, and by the fibrous histotype which is the most aggressive. Each histotype produces different extracellular matrix which affects their migration and spread (Roggli et al, 1992). About 20% of epithelial mesotheliomas synthesize hyaluronan (Roggli et al, 1992) and up to 70% of malignant pleura mesothelioma cases are associated with elevated levels of hyaluronan in the pleura fluid and serum (Thylen et al, 1999). In patients with hyaluronan-producing mesotheliomas, a direct correlation was found between increasing hyaluronan levels in the circulation and tumour burden (Dahl et al, 1989; Thylen et al, 1999). Interestingly, patients responding to theurapeutic treatment exhibited decreased levels of hyaluronan in serum. However, it is hard to make firm statements concerning the relation between hyaluronan-producing mesotheliomas and their clinical aggressiveness due to the limited numbers of patients studied. In this study we have compared the biological properties of the nonhyaluronan-producing mesothelioma cell line Mero-25, with those of the same cells made to produce hyaluronan by transfection of HAS2 cDNA. Increased synthesis of hyaluronan was found to lead to an increased proliferation rate and to anchorage-independent growth in soft agar. The pronounced differencies in the size of colonies formed in soft agar between mock- and HAS2-transfectants indicate an increased malignant phenotype of the hyaluronan-producing mesotheliomas. Attempts to produce tumours after subcutaneous injection of mock- and HAS2-transfectants in nude mice failed (data not shown). This is consistent with previous observations that different mesotheliomas did not produce tumours either injected subcutaneously or intraperitoneally in these animals (Versnel et al, 1989). It is notable, however, that the tumorigenicity of mammary carcinoma and fibrosarcoma cells correlated with their production of hyaluronan (Itano et al, 1999a; Kosaki et al, 1999).

Previous studies suggested that hyaluronan synthesis occurs

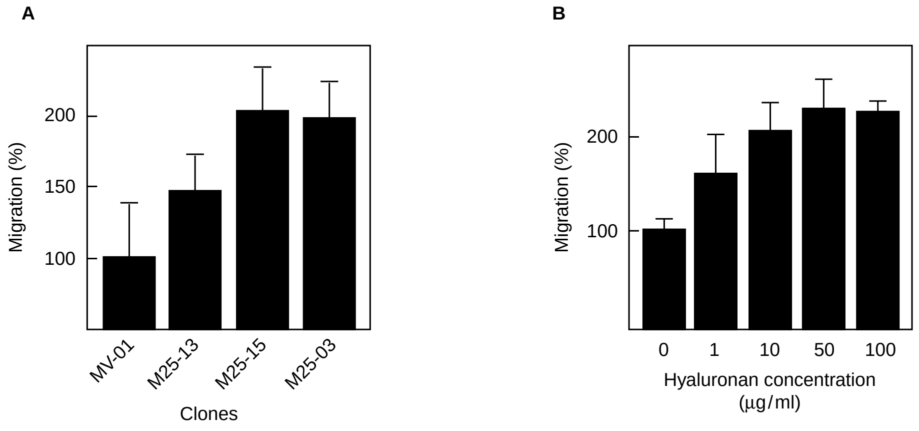

**Figure 6** Effects of hyaluronan on the migration of mesothelioma cells. Migration of mesothelioma transfectants exhibiting different hyaluronan synthesizing capacity was assayed in the Boyden chamber assay, as described in Materials and Methods. Results are represented as the mean ± SEM of 3 separate experiments. (**A**) Migration of mock (MV-01)- and HAS2-transfected mesothelioma cells (M25-13, M25-15, M25-03) in 1% FCS. (**B**) Migration of MV-01 toward different concentrations of hyaluronan

*© 2001 Cancer Research Campaign*

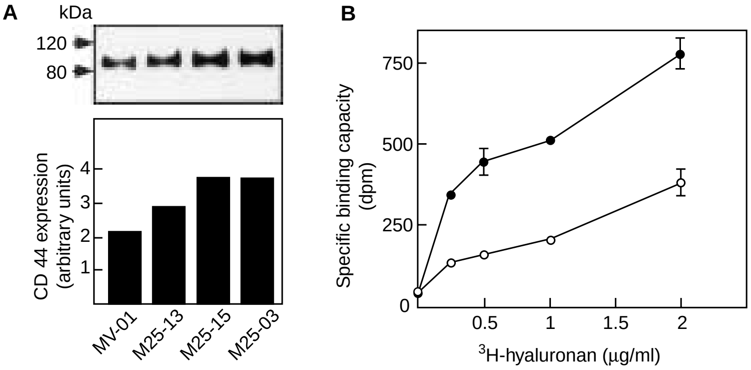

**Figure 7** Expression of CD44 molecules and hyaluronan binding sites on non-hyaluronan-producing and hyaluronan-producing mesothelioma cells. (**A**) Non-hyaluronan-producing (MV-01), and hyaluronan-producing (M25-13, M25-15, M25-03) mesothelioma cells were subjected to immunoblot analysis using mAb Hermes-3 (10 µg $ml^{-1}$). The relative amounts of CD44 molecules were determined by densitometric analysis of the immunoblot. (**B**) Specific cell surface hyaluronan receptors on MV-01 (○–○) and M25-15 (●–●) were detected by incubating the cells with increasing concentrations of [$^3$H]hyaluronan as described in Materials and Methods. Data represent the mean of duplicates ± variation (bars)

during cell division, and probably facilitate transient cell detachment which occurs during mitosis (Brecht et al, 1986). The control mechanism regulating eukaryotic M-phase onset are thought to be triggered by oscillations in the activity of Cdc2 p34 which requires various forms of cyclins; Cdc2 p34-cyclin B plays a critical role in the G2 to M transition whereas Cdc2 p34-cyclin A functions in the S phase (Nurse, 1990). Cell cycle analysis of hyaluronan synthesizing mesothelioma cells revealed a shorter G1 phase and an increase in both S and G2/M compared to nonhyaluronan-producing mesothelioma cells (Figure 5), suggesting that expression of HAS2 gene and protein is associated with the induction of the proliferation markers cyclin A and cyclin B (Figure 4). Furthermore, the visualization of the hyaluronan-containing pericellular matrices around hyaluronan-producing mesothelioma cells may cause the displacement of cellular adhesions molecules thereby facilitating cell detachment and progression through the cell cycle.

During the last few years several studies revealed that CD44-hyaluronan interactions promote invasion and/or metastasis both in vitro and in vivo (Thomas et al, 1992; Naot et al, 1997; Yu et al, 1997; Knudson, 1998). The mechanism proposed to explain how hyaluronan facilitates cell migration is based on its intrinsic physicochemical properties to get hydrated and subsequently induce tissue expansion. The direct tumour cell–hyaluronan interactions mediated through CD44 molecules may play an important role in facilitating haptotactic migration through the tumour-associated hyaluronan-rich matrix. The increase in the expression of CD44 molecules (Figure 7) and the increased migratory ability of HAS2 transfectants (Figure 6), suggests that hyaluronan production by mesothelioma correlates with local invasive behaviour. It is of importance to point out here that cell lines and early-passage mesothelioma cells, but not normal mesothelial cells, express functionally active CD44 hyaluronan receptors (Asplund and Heldin, 1994; Teder et al, 1996). The functional CD44 molecules on mesothelioma could result in an enhanced motility of tumour cells along areas reach in hyaluronan. The molecular mechanisms that control the gene expression of the three HAS isoforms and their significance in terms of the function of hyaluronan synthesized has not been fully elucidated so far. According to our and other laboratories observations (Brinck and Heldin, 1999; Itano et al, 1999b) each HAS isoform exhibit intrinsic differencies both in their expression pattern and in the amount and size of the synthesized polymer. A more recent report revealed that the HAS2 isoform was induced during the healing of a mechanical injury of mesothelial cell cultures and that hyaluronan synthesis was located at the leading edge of the wound and promoted cell migration (Yung et al, 2000). However, concerning the role of hyaluronan in cell migration, further studies by us (Brinck and Heldin, 1999) and other laboratories (Forrester and Wilkinson, 1981) revealed an inverse correlation between cell migration and hyaluronan levels surrounding the cells. It should be emphasized, however, that other cell types were investigated. In addition, the HAS3 isoform, and not the HAS2 isoform, was overexpressed (Brinck and Heldin, 1999). Taken together, most likely hyaluronan synthesized by each one of the 3 HAS isoforms affects cell migration in a manner which is dependent on polymer size, concentration and cell target type. Immunohistochemical studies of malignant mesotheliomas producing or not producing hyaluronan revealed that hyaluronan production correlated to the expression of different antigens most likely indicating a more anaplastic tumour (Thylén et al, 1997). An elucidation of the mechanisms involved in the induction of hyaluronan synthesis by mesotheliomas will increase our understanding of the parameters which influence local spreading of malignant mesothelioma. Such knowledge will be important when considering future therapeutic strategies of mesothelioma.

### ACKNOWLEDGEMENTS

We would like to thank professor TC Laurent for constructive criticism of this work and Dr Nils-Erik Heldin for valuable discussions on the cell cycle. The excellent technical assistance of M Svarvare and A Hermansson is also gratefully acknowledged. This work was supported by grants from the Swedish Cancer Society (3446-896-03XBB), the Swedish Medical Research Council (K97-03X), King Gustav V:s 80-års fond, Göran Gustafsson Foundation, Wenner-Gren Foundation and Q -Med AB.

### REFERENCES

Asplund T and Heldin P (1994) Hyaluronan receptors are expressed on human malignant mesothelioma cells but not on normal mesothelial cells. *Cancer Res* **54**: 4516–4523

Asplund T, Versnel MA, Laurent TC and Heldin P (1993) Human mesothelioma cells produce factors that stimulate the production of hyaluronan by mesothelial cells and fibroblasts. *Cancer Res* **53**: 388–392

Auvinen PK, Parkkinen JJ, Johansson RT, Ågren UM, Tammi RH, Eskelinen MJ and

© 2001 Cancer Research Campaign

Kosma VM (1997) Expression of hyaluronan in benign and malignant breast lesions. *Int J Cancer* **74**: 477–481

Bertrand P, Girard N, Delpech B, Duval C, d'Anjou J and Dauce JP (1992) Hyaluronan (hyaluronic acid) and hyaluronectin in the extracellular matrix of human breast carcinomas: comparison between invasive and non-invasive areas. *Int J Cancer* **52**: 1–6

Bielefeldt-Ohmann H, Jarnicki AC and Fitzpatrick DR (1996) Molecular pathobiology and immunology of malignant mesothelioma. *J Pathol* **178**: 369–378

Brecht M, Mayer U, Schlosser E and Prehm P (1986) Increased hyaluronate synthesis is required for fibroblast detachment and mitosis. *Biochem J* **239**: 445–450

Brinck J and Heldin P (1999) Expression of Recombinant Hyaluronan Synthase (HAS) Isoforms in CHO Cells Reduces Cell Migration and Cell Surface CD44. *Exp Cell Res* **252**: 342–351

Clarris BJ and Fraser JRE (1968) On the pericellular zone of some mammalian cells *in vitro Exp Cell Res* **49**: 181–193

Dahl IMS, Solheim ØP, Erikstein B and Müller E (1989) A longitudinal study of the hyaluronan level in the serum of patients with malignant mesothelioma under treatment. Hyaluronan as an indicator of progressive disease. *Cancer* **64**: 68–73

Delpech B, Girard N, Olivier A, Maingonnat C, van Driessche G, van Beeumen J, Bertrand P, Duval C, Delpech A and Bourguignon J (1997) The origin of hyaluronectin in human tumors. *Int J Cancer* **72**: 942–948

Forrester JV and Wilkinson PC (1981) Inhibition of leukocyte locomotion by hyaluronic acid. *J Cell Sci* **48**: 315–331

Freedman VH and Shin S (1974) Cellular tumorigenicity in nude mice: correlation with cell growth in semi-solid medium. *Cell* **3**: 355–359

Frizelle SP, Grim J, Zhou J, Gupta P, Curiel DT, Geradts J and Kratzke RA (1998) Re-expression of p16INK4a in mesothelioma cells results in cell cycle arrest, cell death, tumor suppression and tumor regression. *Oncogene* **16**: 3087–3095

Heldin P and Pertoft H (1993) Synthesis and assembly of the hyaluronan-containing coats around normal human mesothelial cells. *Exp Cell Res* **208**: 422–429

Heldin P, Suzuki M, Teder P and Pertoft H (1995) Chondroitin sulfate proteoglycan modulates the permeability of hyaluronan-containing coats around normal human mesothelial cells. *J Cell Physiol* **165**: 54–61

Itano N and Kimata K (1996) Molecular cloning of human hyaluronan synthase. *Biochem Biophys Res Commun* **222**: 816–820

Itano N, Sawai T, Miyaishi O and Kimata K (199a) Relationship between hyaluronan production and metastatic potential of mouse mammary carcinoma cells. *Cancer Res* **59**: 2499–2504

Itano N, Sawai T, Yoshida M, Lenas P, Yamada Y, Imagawa M, Shinomura T, Hamaguchi M, Yoshida Y, Ohnuki Y, Miyauchi S, Spicer AP, McDonald JA and Kimata K (1999b) Three isoforms of mammalian hyaluronan synthases have distinct enzymatic properties. *J Biol Chem* **274**: 25085–25092

Jacobson A, Brinck J, Briskin MJ, Spicer AP and Heldin P (2000) Expression of human hyaluronan synthases in response to external stimuli. *Biochem J* **348**: 29–35

Kimata K, Honma Y, Okayama M, Oguri K, Hozumi M and Suzuki S (1983) Increased synthesis of hyaluronic acid by mouse mammary carcinoma cell variants with high metastatic potential. *Cancer Res* **43**: 1347–1354

Klominek J, Robért K-H, Hjerpe A, Wickström B and Gahrton G (1989) Serum-dependent growth patterns of two, newly established human mesothelioma cell lines. *Cancer Res* **49**: 6118–6122

Knudson W (1998) The role of CD44 as a cell surface hyaluronan receptor during tumor invasion of connective tissue. *Front Bioscience* **3**: D604–D615

Kosaki R, Watanabe K and Yamaguchi Y (1999) Overproduction of hyaluronan by expression of the hyaluronan synthase Has2 enhances anchorage-independent growth and tumorigenesis. *Cancer Res* **59**: 1141–1145

Laurent TC and Fraser JRE (1992) Hyaluronan. *FASEB J* **6**: 2397–2404

Laurent TC, Laurent UBG and Fraser JRE (1995) Functions of hyaluronan. *Ann Rheum Dis* **54**: 429–432

Lebel L, Smith L, Risberg B, Gerdin B and Laurent TC (1988) Effect of increased hydrostatic pressure on lymphatic elimination of hyaluronan from sheep lung. *J Appl Physiol* **64**: 1327–1332

Li Y, Rahmanian M, Widström C, Lepperdinger G, Frost GI and Heldin P (2000) Irradiation-induced expression of hyaluronan (HA) synthase 2 and hyaluronidase 2 genes in rat lung tissue accompanies active turnover of HA and Induction of Types I and III Collagen gene expression. *Am J Respir Cell Mol Biol* **23**: 411–418

Metcalf RA, Welsh JA, Bennett WP, Seddon MB, Lehman TA, Pelin K, Linnainmaa K, Tammilehto L, Mattson K and Gerwin BI (1992) p53 and Kirsten-ras mutations in human mesothelioma cell lines. *Cancer Res* **52**: 2610–2615

Naot D, sionov RV and Ish-shalom D (1997) CD44: structure, function, and association with the malignant process. In *Adv Cancer Res* Vande Woude G F and Klein G (eds), Vol. 71. pp. 241–319

Nurse P (1990) Universal control mechanism regulating onset of M-phase. *Nature* **344**: 503–507

Roggli VL, D.S. G and Pratt PC (1992) *Pathology of Asbestos-Associated Diseases*. Little Brown: Boston

Ropponen K, Tammi M, Parkkinen J, Eskelinen M, Tammi R, Lipponen P, Ågren U, Alhava E and Kosma VM (1998) Tumor cell-associated hyaluronan as an unfavorable prognostic factor in colorectal cancer. *Cancer Res* **58**: 342–347

Scarpa S, Giuffrida A, Fazi M, Coletti A, Palumbo C, Pass HI, Procopio A and Modesti A (1999) Migration of mesothelioma cells correlates with histotype-specific synthesis of extracellular matrix. *Int J Mol Med* **4**: 67–71

Shyjan AM, Heldin P, Butcher EC, Yoshino T and Briskin MJ (1996) Functional cloning of the cDNA for human hyaluronan synthase. *J Biol Chem* **271**: 23395–23399

Spicer AP, Augustine ML and McDonald JA (1996) Molecular cloning and characterization of a putative mouse hyaluronan synthase. *J Biol Chem* **271**: 23400–23406

Spicer AP, Olson JS and McDonald JA (1997) Molecular cloning and characterization of a cDNA encoding the third putative mammalian hyaluronan synthase. *J Biol Chem* **272**: 8957–8961

Teder P, Versnel MA and Heldin P (1996) Stimulatory effects of pleural fluids from mesothelioma patients on CD44 expression, hyaluronan production and cell proliferation in primary cultures of normal mesothelial and transformed cells. *Int J Cancer* **67**: 393–398

Tengblad A (1980) Quantitative analysis of hyaluronan in nanogram amounts. *Biochem J* **185**: 101–105

Thomas L, Byers HR, Vink J and Stamenkovic I (1992) CD44H regulates tumor cell migration on hyaluronan-coated substrate. *J Cell Biol* **118**: 971–977

Thylén A, Levin-Jacobsen A-M, Hjerpe A and Martensson G (1997) Immunohistochemical differences between hyaluronan- and non-hyaluronan-producing malignant mesothelioma. *Eur Respir J* **10**: 404–408

Thylen A, Wallin J and Martensson G (1999) Hyaluronan in serum as an indicator of progressive disease in hyaluronan-producing malignant mesothelioma. *Cancer* **86**: 2000–2005

Versnel MA, Bouts MJ, Hoogsteden HC, Van der Kwast TH, Delahaye M and Hagemeijer A (1989) Establishment of human malignant mesothelioma cell lines. *Intern J Cancer* **44**: 256–260

Vindelöv LL, Christensen IJ and Nissen NI (1983) A detergent-trypsin method for the preparation of nuclei for flow cytometric DNA analysis. *Cytometry* **3**: 323–327

Watanabe K and Yamaguchi Y (1996) Molecular identification of a putative human hyaluronan synthase. *J Biol Chem* **271**: 22945–22948

Yu Q, Toole BP and Stamenkovic I (1997) Induction of apoptosis of metastatic mammary carcinoma cells in vivo by disruption of tumor cell surface CD44 function. *J Exp Med* **186**: 1985–1996

Zhang L, Underhill CB and Chen L (1995) Hyaluronan on the surface of tumor cells is correlated with metastatic behavior. *Cancer Res* **55**: 428–433

© 2001 Cancer Research Campaign
